# RRM2 protects against ferroptosis and is a tumor biomarker for liver cancer

**DOI:** 10.1186/s12935-020-01689-8

**Published:** 2020-12-07

**Authors:** Yueyue Yang, Jiafei Lin, Susu Guo, Xiangfei Xue, Yikun Wang, Shiyu Qiu, Jiangtao Cui, Lifang Ma, Xiao Zhang, Jiayi Wang

**Affiliations:** 1 Department of Clinical Laboratory, Shanghai Tenth People’s Hospital of Tongji University, Shanghai, 200072 China; 2 Department of Clinical Laboratory, Ruijin Hospital, Shanghai Jiao Tong University School of Medicine, Shanghai, 200025 China; 3 Shanghai Institute of Thoracic Oncology, Shanghai Chest Hospital, Shanghai Jiao Tong University, Shanghai, 200030 China; 4 Department of Thoracic Surgery, Shanghai Institute of Thoracic Tumors, Shanghai Chest Hospital, Shanghai Jiao Tong University, No. 241 Huaihai West Road, Shanghai, 200030 China

**Keywords:** GSS, Proteasome, Diagnosis, Phosphorylation, Glutathione, Erastin

## Abstract

**Background:**

Ferroptosis is the process of cell death triggered by lipid peroxides, and inhibition of glutathione (GSH) synthesis leads to ferroptosis. Liver cancer progression is closely linked to ferroptosis suppression. However, the mechanism by which inhibition of GSH synthesis suppresses potential ferroptosis of liver cancer cells and whether ferroptosis-related liver cancer biomarkers have a promising diagnostic value remain unknown.

**Methods:**

Ribonucleotide reductase regulatory subunit M2 (RRM2) levels were measured using an enzyme linked immunosorbent assay (ELISA), quantitative RT-PCR (qPCR), immunoblotting (IB) and immunochemistry (IHC). Cell viability and cell death were measured by a CellTiter-Glo luminescent cell viability assay and staining with SYTOX Green followed by flow cytometry, respectively. Metabolites were measured using the indicated kits. The Interaction between glutathione synthetase (GSS) and RRM2 was measured using immunofluorescence (IF), co-immunoprecipitation (co-IP) and the proximal ligation assay (PLA). The diagnostic value was analyzed using the area under the receiver operating characteristic curve (AUC-ROC). Bioinformatics analysis was performed using the indicated database.

**Results:**

RRM2 showed specifically elevated levels in liver cancer and inhibited ferroptosis by stimulating GSH synthesis via GSS. Mechanistically, phosphorylation of RRM2 at the Threonine 33 residue (T33) was maintained at normal levels to block the RRM2–GSS interaction and therefore protected RRM2 and GSS from further proteasome degradation. However, under ferroptotic stress, RRM2 was dephosphorylated at T33, thus the RRM2–GSS interaction was promoted. This resulted in the translocation of RRM2 and GSS to the proteasome for simultaneous degradation. Clinically, serum RRM2 was significantly associated with serum alpha-fetoprotein (AFP), carcinoembryonic antigen (CEA), alanine aminotransferase (ALT), aspartate aminotransferase (AST), alkaline phosphatase (ALP), gamma glutamyl transpeptidase (γ-GT), albumin (ALB) and total bilirubin. The AUC-ROC for the combination of RRM2 with AFP was 0.947, with a sensitivity of 88.7% and a specificity of 97.0%, which indicates better diagnostic performance compared to either RRM2 or AFP alone.

**Conclusion:**

RRM2 exerts an anti-ferroptotic role in liver cancer cells by sustaining GSH synthesis. Serum RRM2 will be useful as a biomarker to evaluate the degree to which ferroptosis is suppressed and improve diagnostic efficiency for liver cancer.

## Background

Cell death is the ultimate fate of all cells and has an irreplaceable role in the entire body in a manner similar to cell division and proliferation [1]. Through investigating of cell death, one would know the process of tumor initiation and development, and propose new treatments and diagnoses for cancers [2]. Well-established cell death processes include apoptosis, necrosis, paraptosis and autophagy [3–5]. A new type of regulated cell death named ferroptosis was discovered by Dixon et al. [6], and the accumulation of lipid reactive oxygen species (ROS) is one of its hallmarks. Emerging studies have delineated that the agonists of ferroptosis can directly or indirectly impair the antioxidant glutathione (GSH) through different pathways, resulting in excessive aggregation of lipid ROS and, ultimately, cell death [7]. The close relationships between ferroptosis and various diseases, such as Huntington’s disease [8], ischemia–reperfusion injury [9] and kidney injury [10], have been gradually recognized. Additionally, ferroptosis has been linked with malignant diseases, such as diffuse large B-cell lymphoma [11], renal cell carcinoma [11], ovarian cancer [12], osteosarcoma [13] and prostate adenocarcinoma [13].

Liver cancer is the second leading cause of cancer-related death worldwide [14, 15]. However, treatment options, including surgical resection, transplantation and molecular drug therapies, are of limited effectiveness [16]. Recent studies have demonstrated that suppressing ferroptosis might be a pivotal signal for liver cancer initiation [17–19], thus providing a new way to combat liver cancer. Moreover, a transcriptional regulatory network has been identified in liver cancer cells, in which the transcription factor (TF) hepatocyte nuclear factor 4 alpha (HNF4A) modulates the transcription of a series of anti-ferroptotic molecules [18]. Other examples from the p62–Keap1–NRF2 pathway and metallothionein-1G (MT-1G) suggest that ferroptosis is strictly inhibited in liver cancer cells [20, 21]. However, endogenous ferroptosis suppressors in liver cancer cells are still far from known.

To date, the majority of patients with liver cancer are diagnosed at the middle-late stage, and the sensitivity and specificity of liver cancer biomarkers are not very satisfactory [22–24]. The vast majority of studies have demonstrated that using a panel of biomarkers in addition to classic alpha fetoprotein (AFP) definitely increases diagnostic accuracy and sensitivity [25–27]. Given that suppressing ferroptosis is closely correlated with liver tumorigenesis, serum biomarkers that reflect ferroptosis inhibition may have potential diagnostic efficiency. However, the discovery and verification of such liver cancer biomarkers are still lacking.

Ribonucleotide reductase (RR) is a structural unit required for DNA replication and repair [28]. RR consists of two subunits namely RRM1 and RRM2. RRM1 shows relatively constant protein expression throughout the whole life of a cell, whereas RRM2 protein expression dynamically changes upon stimulation [28, 29]. RRM2 has been proven to participate in the regulation and modification of proteins [30–32]. RRM2 is also considered a vital component in tumor progression [33], a regulator of some oncogenes [34] and a promising tumor biomarker for many cancers [35, 36]. RRM2 antagonizes sorafenib, an FDA-approved multikinase inhibitor, to treat liver cancer, possibly due to its function to partially rescue liver cancer cells from sorafenib-induced long-term cytotoxicity [37]. Recently, the roles of sorafenib in triggering ferroptosis have been established in liver cancer cells [38, 39]. However, whether and how RRM2 protects liver cancer cells against ferroptosis is still not known. Additionally, the potential usage of serum RRM2 as a biomarker to diagnose liver cancer remains unclear.

Therefore, we investigated whether RRM2 acts as a potential target to suppress ferroptosis in liver cancer cells. The potential diagnostic value of serum RRM2 to predict liver cancer was also evaluated. We discovered that RRM2 exhibits protumorigenic activity in liver cancer. The overexpression of RRM2 is linked with tumor progression in liver cancer. Herein, through our investigations, we also proposed that RRM2 is an endogenous ferroptosis suppressor that sustains the expression of glutathione synthetase (GSS), which is critical for GSH synthesis. Of note, we provided further evidence that serum RRM2 is a promising biomarker for the diagnosis of liver cancer. Taken together, these findings indicate that proteins that protect against ferroptosis can be regarded as both targets and biomarkers for the treatment and diagnosis of liver cancer.

## Methods

### Patients and blood samples

In all, 185 patients (120 men and 65 women, age range 37–78 years) were diagnosed with liver cancer via enhanced computed tomography (CT), magnetic resonance imaging (MRI) and ultrasonic-guided biopsy analysis at Shanghai Ruijin Hospital and Shanghai Tenth People's Hospital. There were 141 patients diagnosed with chronic hepatitis by serological or pathological examination at Shanghai Ruijin Hospital, including 83 patients with hepatitis A, 32 patients with hepatitis B and 26 patients with hepatitis C. There were 103 patients diagnosed with malignant tumors via pathological examination at Shanghai Ruijin Hospital, including 24 patients with gastric cancer, 25 patients with breast cancer, 29 patients with colorectal cancer and 25 patients with lung cancer. There were 100 healthy volunteers with no history of liver diseases or alcoholism at Shanghai Ruijin Hospital and Shanghai Tenth People's Hospital. After a 5 ml of venous blood was collected from each patient and healthy volunteer, it was centrifuged for 10 min at 3,000 rpm at 4 °C. All the plasma samples normally frozen at − 80 °C were completely thawed to room temperature before they were tested. Our protocol was approved by the institutional review boards of Shanghai Ruijin Hospital and Shanghai Tenth People's Hospital and written informed consent was obtained from each patient and healthy volunteer. All experiments were carried out in accordance with the Declaration of Helsinki.

### Cell culture and vectors

The cell lines used in this study are as follows: hepatocyte line HL-7702 (Cell Bank of Chinese Academy of Sciences, Shanghai, China), and liver cancer cell lines Bel-7402 (Cell Bank of Chinese Academy of Sciences), SMMC-7721 (Cell Bank of Chinese Academy of Sciences), SK-Hep-1 (Cell Bank of Chinese Academy of Sciences), Huh-7 (Cobier, Nanjing, China), Bel-7404 (Cell Bank of Chinese Academy of Sciences) and HepG2 (Cobier). All cell lines were cultured in Dulbecco’s modified Eagle’s medium (DMEM) supplemented with 10% fetal bovine serum (Gibco, Carlsbad, CA, USA) and 1% penicillin and streptomycin (Gibco). Erastin (Sigma, St Louis, MO, USA), MG132 (MedChemExpress, Monmouth Junction, NJ, USA), ferrostatin-1 (Fer-1) (Sigma), ZVAD-FMK (Sigma), necrostatin-1 (Nec-1) (Sigma), NU6102 (Sigma) and SB203580 (Sigma) were used to treat the cells.

RRM2 overexpression and RRM2^sh1^ knockdown plasmids were obtained from Origene (Beijing, China). RRM2^sh2^ plasmid was purchased from Biolink (Shanghai, China). RRM2^T33A^ and RRM2^T33E^ plasmids were constructed using overlapping PCR. LentiCRISPR v2-based constructs were used to knockout of RRM2 and GSS. The primers and sgRNAs used were listed in Additional file 1: Table S1.

### Immunoblotting (IB)

The proteins were resolved on SDS–PAGE gels with or without phos-tag™ reagents (Dako, Kyoto, Japan) according to the standard protocol. The primary antibodies were: anti-GAPDH (Cell Signaling Technology (CST), Boston, MA, USA, #5174 or #51332), anti-RRM2 (Abcam, Hong Kong, China, #ab172476 and #ab57653), anti-CBS (Abcam, #ab140600), anti-CTH (Abcam, #ab189916), anti-SHMT2 (Abcam, #ab180786), anti-GPX4 (Abcam, #ab125066), anti-GSS (Abcam, #ab124811, or Sigma, #SAB1403888), anti-ALB (Abcam, #ab207327), anti-Myc (CST, #2276 or #2278) and anti-PSMB5 (Abcam, #ab167341 or #ab3330).

### Immunohistochemistry (IHC) and immunofluorescence (IF)

IHC and IF were performed using conventional protocols that are available elsewhere. For IHC, the primary antibody used was anti-RRM2 (Abcam, #ab172476). The tissue microarray was purchased from U.S. Biomax (Rockville, MD, USA). The final results were confirmed by two independent pathologists. The specimens were scored as follows: the one in which 0% of cells showing signals for staining was tagged as negative (−), 0–10% of cells was tagged as weak positive (−/+) and 11–100% of cells was tagged as strong positive (+). For IF, the primary antibodies used were anti-GSS (Abcam, #ab124811), anti-PSMB5 (Abcam, # ab167341) and anti-RRM2 (Abcam, #ab172476).

### Evaluation of cell viability and cell death

Cell viability was measured using a CellTiter-Glo luminescent cell viability assay (Promega, Madison, WI, USA) according to the manufacturer’s instructions. Cell death was analyzed by staining with SYTOX Green (Invitrogen, Carbsland, CA, USA) followed by flow cytometry.

### Enzyme-linked immunosorbent assay (ELISA) and measurements of metabolites

ELISA kits for the detection of RRM2, AFP, glycine, glutamate, cysteine, cystathionine and serine were purchased from Lichen Biotech Ltd. (Shanghai, China). ELISAs were performed strictly in accordance with guidelines provided by manufacturer. Labile iron and 4-HNE were measured using kits from Abcam. GSH and phospholipid level were measured using kits from Sigma.

### Quantitative RT-PCR (qPCR)

Total RNA was extracted using TRIzol reagent (Ambion, Carlsbad, CA, USA) according to the manufacturer’s instruction and subjected to the PrimeScript RT Reagent Kit (TaKaRa, Dalian, China). The resultant RNA was used to evaluate the expression of relevant proteins. The primers used were listed in Additional file 1: Table S1.

### Co-immunoprecipitation (co-IP)

Cell lysates were incubated with antibodies at 4 °C overnight, followed by incubation with protein A/G magnetic beads (Thermo Scientific, Waltham, MA, USA). Subsequently, the beads were collected and subjected to IB. The antibodies used for co-IP were anti-Myc (CST, #2276), anti-RRM2 (Abcam, #ab172476), anti-PSMB5 (Abcam, #ab167341) and anti-GSS (Abcam, #ab124811).

### Proteasome isolation

Proteasomes were isolated using the proteasome isolation kit from Sigma (#539176). Isolation was performed strictly in accordance with guidelines provided by the manufacturer. Affinity and control beads were used to isolate the proteasome and serve as a negative control.

### Proximity ligation assay (PLA)

The PLA was performed using a Duolink™ proximity ligation kit from Sigma. Briefly, cells subjected to specific treatments were seeded on glass cover slips in 24-well plates. On the second day, cells were fixed with 4% PFA and blocked with the blocking buffer. Next, the cells were incubated overnight at 4 °C with suitable primary antibodies. The primary antibodies used were: anti-Myc (CST, #2276) and anti-GSS (Abcam, #ab124811). On the third day, the PLA probe solution was added into each well and incubated for 1 h at 37 °C, followed by the addition of Ligase-Ligase solution into each well and incubation for 30 min at 37 °C. After ligation, the amplification-polymerase solution was added into each well and incubated for 100 min at 37 °C before the cells were subjected to microscopic analysis.

### Bioinformatics analysis

Tandem mass tag (TMT) data (ProteomeXchange Consortium: PXD010761), RNA-seq data (GEO database: GSE104462) and DNAse-seq data (ENCODE database: ENCSR149XIL and ENCSR555QAY) were used in our previous study [18]. Data mining from The Cancer Genome Atlas (TCGA) was performed by Luming Biotechnology (Shanghai, China). Protein localization was analyzed in the UniProt database (https://www.uniprot.org). RRM2 expression in tumor and normal tissues from corresponding organs was analyzed in the UALCAN database (http://ualcan.path.uab.edu). The correlation between RRM2 expression and overall survival of liver cancer patients was analyzed in the KM plotter database (https://kmplot.com/analysis).

### Statistical analysis

Tests used to examine the differences between groups were Student’s t test, one-way ANOVA, χ^2^ test and Spearman rank-correlation analysis. P < 0.05 was considered statistically significant. The area under the receiver operating characteristic curve (AUC-ROC) analysis was performed to find the diagnostic values of serum RRM2 and AFP both alone and in combination of RRM2 and AFP for the prediction of liver cancer.

## Results

### RRM2 is associated with ferroptosis, and its upregulation in liver cancer is linked with a poor clinical outcome

**Fig. 1:**
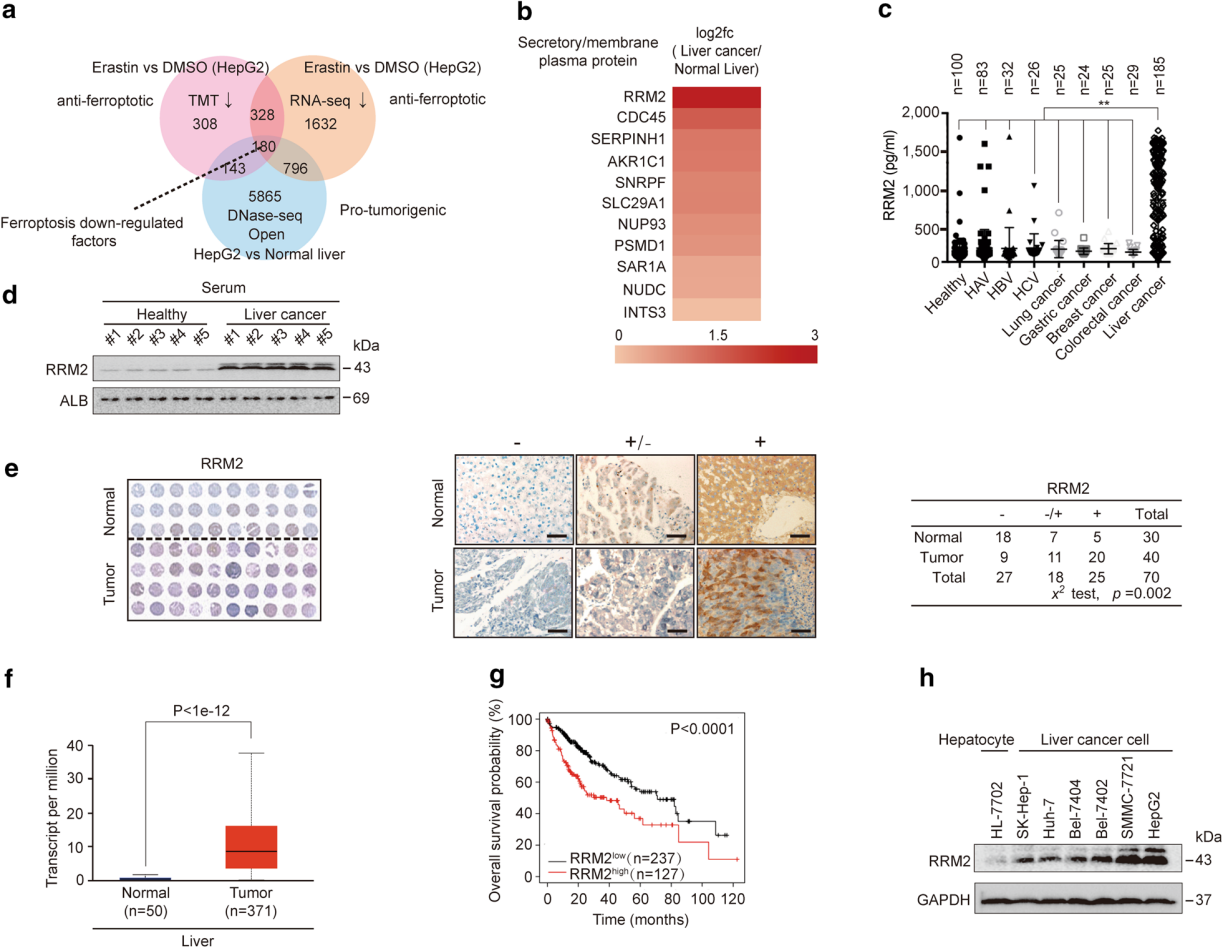
RRM2 is highly expressed in liver cancer. **a** The 180 ferroptosis-related factors that were downregulated were identified by TMT, RNA-seq and DNase-seq. **b** RRM2 was identified as the most significantly upregulated secretory or membrane-bound protein in liver cancer via data mining using the TCGA database. **c** Scatter plot for serum RRM2 in healthy individuals and patients with hepatitis A, hepatitis B, hepatitis C, lung cancer, gastric cancer, breast cancer, colorectal cancer or liver cancer. **d** RRM2 expression in the sera from healthy individuals and liver cancer patients, as was evaluated by immunoblotting. **e** TMA of RRM2 in liver cancer and normal liver tissues. Representative IHC images of TMA stained with anti-RRM2 antibodies are shown. Data were analyzed using a chi-square test. **f** The UALCAN database was used to analyze alterations in RRM2 expression between liver cancer (n = 371) and normal liver (n = 50) tissues. **g** Kaplan–Meier survival plots of RRM2 were obtained from the KM plotter database. **h** RRM2 was highly expressed in SMMC-7721 and HepG2 cell. RRM2 expression was measured by immunoblotting with anti-RRM2 antibodies in established hepatocyte (HL-7702) and liver cancer cell lines, as indicated. The IB data are representative images from three biological replicates. **P < 0.01 indicates statistical significance. Data in c were analyzed using a one-way ANOVA test. Data in e were analyzed using a chi-square test. Data in f were analyzed using Student’s t test. Data in g were analyzed using log rank analysis

Because anti-ferroptotic factors are usually suppressed under ferroptotic stress [17, 18], we first sought potential factors, that were downregulated following treatment with erastin, a well-established ferroptosis agonist, by utilizing proteomics and transcriptomics data from a previous study of ours [18]. Through TMT and RNA-seq, 508 factors that showed downregulation of both mRNA and protein levels by erastin were identified (Fig. 1a). By further comparing DNase-seq data from the liver cancer cell line HepG2 (ENCODE database: ENCSR149XIL) and normal liver (ENCODE database: ENCSR555QAY), more obvious open chromatins were observed within the promoters of 180 out of those 508 factors in HepG2 cells compared to normal liver (Fig. 1a), suggesting that these 180 downregulated ferroptosis factors might be also protumorigenic. Secretory/membrane proteins are potential biomarkers because they are more easily released into the bloodstream [40–42]. By data mining from the TCGA database and combining these data with protein localization information from UniProt (https://www.uniprot.org), RRM2 was identified as the most significantly upregulated secretory/membrane protein among the 180 candidates (Fig. 1b). As expected, RRM2 could be detected in serum, and its concentration was remarkably elevated in patients bearing liver cancer compared to that in healthy individuals and in patients with hepatitis A, B and C, and other malignancies, including primary lung, gastric, breast and colorectal cancers (Fig. 1c, d), suggesting that serum RRM2 is a potential biomarker to diagnose liver cancer.

By testing 30 normal liver and 40 liver cancer tissue specimens using tissue microarray assay (TMA), RRM2 was further confirmed to be significantly upregulated in liver cancer compared to normal liver (Fig. 1e), which is supported by the data from the UALCAN database (http://ualcan.path.uab.edu) (Fig. 1f). Additionally, higher RRM2 expression in liver cancer signifies poorer overall survival (Fig. 1g). In addition, higher levels of RRM2 were detected in liver cancer cell lines (SK-Hep-1, Huh-7, Bel-7404, Bel-7402, SMMC-7721, HepG2) than in the hepatocyte line HL-7702 (Fig. 1h), suggesting the potential role of RRM2 in promoting liver tumorigenesis. Since HepG2 and SMMC-7721 cells were shown to exhibit high carcinogenic properties in our previous studies [18, 43–45] and had the highest levels of RRM2 among the liver cancer cell lines tested, we thereby chose these two liver cancer cell lines as the main materials in subsequent experiments.

### RRM2 is an endogenous ferroptosis inhibitor that elevates GSH

**Fig. 2:**
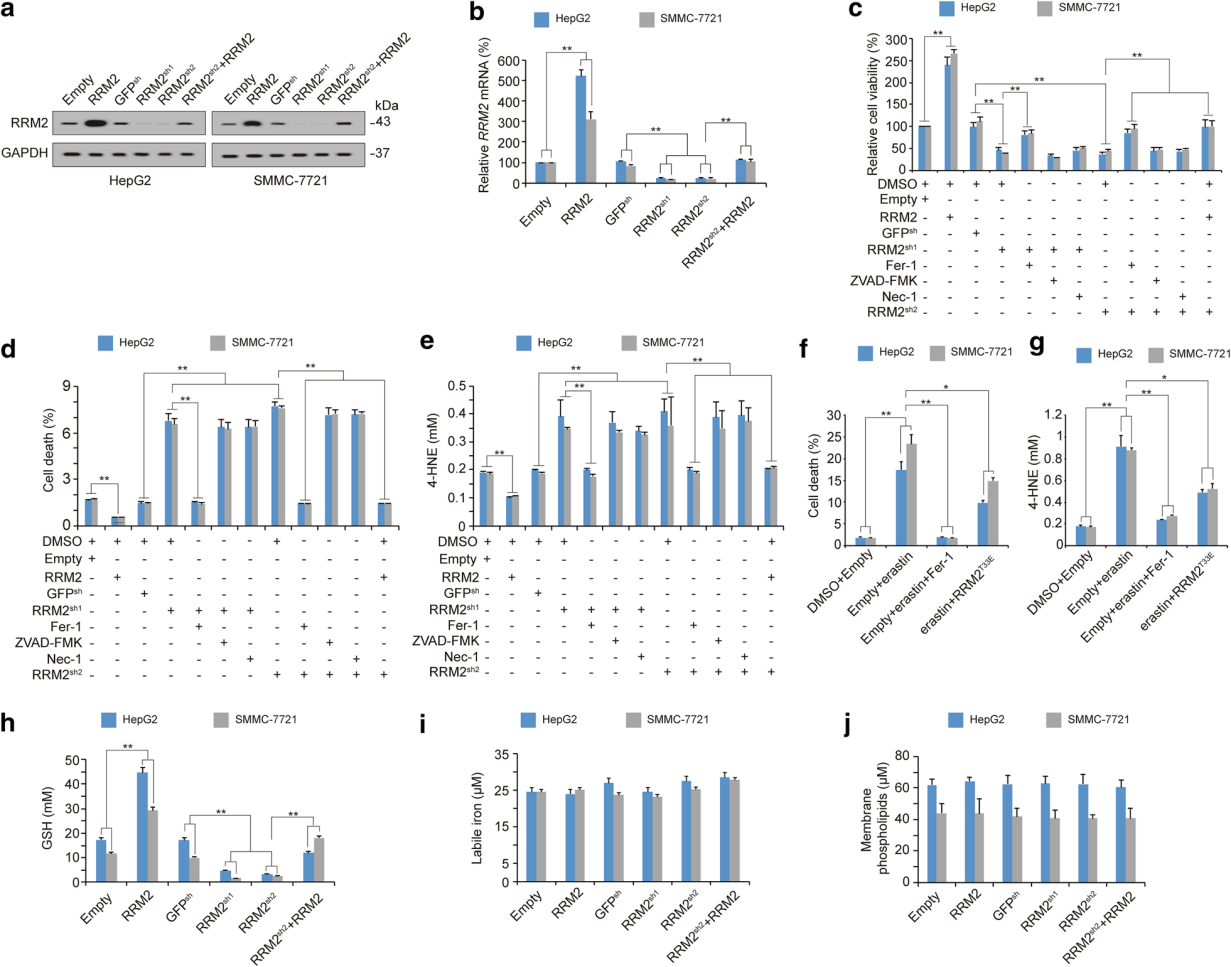
RRM2 suppresses ferroptosis in liver cancer cells. **a**, **b** RRM2 protein (**a**) and mRNA levels (**b**) in HepG2 and SMMC-7721 cells with or without RRM2 overexpression or knockdown, as analyzed by immunoblotting and qPCR, respectively. **c**–**e** Cell viability (**c**), cell death (**d**) and 4-HNE levels (**e**) were measured in HepG2 and SMMC-7721 cells with ectopically expressed or knocked down RRM2 before further treatment with Fer-1, ZVAD-FMK, Nec-1 or ectopically expressed RRM2. Cell viability was measured using a CellTiter-Glo luminescent cell viability assay, cell death was measured by staining with SYTOX Green followed by flow cytometry, and 4-HNE was measured by a kit from Abcam. **f**, **g** Cell death (**f**) and 4-HNE levels (**g**) were measured in HepG2 and SMMC-7721 cells treated with erastin (10 μM, 24 h) in the presence or absence of Fer-1 (1 μM, 24 h). The cells were also cultured with or without RRM2^T33E^ transfection, as indicated. **h**–**j** The levels of GSH (**h**), labile iron (**i**) and membrane-anchored phospholipids (**j**) in HepG2 and SMMC-7721 cells with or without RRM2 overexpression or knockdown were analyzed. The data are shown as the mean ± SD from three biological replicates (including IB). *P < 0.05, **P < 0.01 indicates statistical significance. Data from **b**−**j** were analyzed using a one-way ANOVA test

Subsequently, we evaluated whether RRM2 suppresses ferroptosis in liver cancer cells. By introducing exogenous RRM2 or two independent shRNAs targeting RRM2 into HepG2 and SMMC-7721 cells (Fig. 2a, b), we found that RRM2 overexpression promoted cell viability, whereas RRM2 knockdown inhibited cell viability (Fig. 2c). RRM2^sh2^ was specifically designed to target the 3′UTR of *RRM2* mRNA; therefore, its inhibitory role on cell viability could be reversed by simultaneously overexpressing RRM2 (Fig. 2c). The inhibitory effects on RRM2 knockdown could also be reversed by ferrostatin-1 (Fer-1, a ferroptosis inhibitor) but not be influenced by ZVAD-FMK (an apoptosis inhibitor) or necrostatin-1 (Nec-1, a necrosis inhibitor) (Fig. 2c), suggesting that RRM2 depletion-impaired cell viability is associated with ferroptosis. Cell death and 4-HNE, a product of lipid peroxidation, were also evaluated, and we found that RRM2 has the capacity to suppress ferroptosis and ferroptosis-associated 4-HNE generation (Fig. 2d, e), indicating that RRM2 is an endogenous ferroptosis inhibitor. Because RRM2 was downregulated following erastin treatment (Fig. 1a), we wondered whether supplementation with RRM2 could reverse erastin-induced ferroptosis. Indeed, erastin-resistant RRM2^T33E^ (will be discussed in the following section) partially rescued erastin-induced ferroptosis and ferroptosis-associated 4-HNE generation (Fig. 2f, g).

The accumulation of lipid ROS is regarded as the final step in inducing ferroptosis [46, 47]. At least three metabolites, including labile iron, membrane-anchored phospholipids and GSH, control the generation of lipid ROS [6, 18, 48]. However, only GSH was found to be positively regulated by RRM2 (Fig. 2h–j), suggesting that RRM2 suppresses ferroptosis by elevating the level of GSH.

### GSS is required for RRM2 to increase GSH levels

**Fig. 3:**
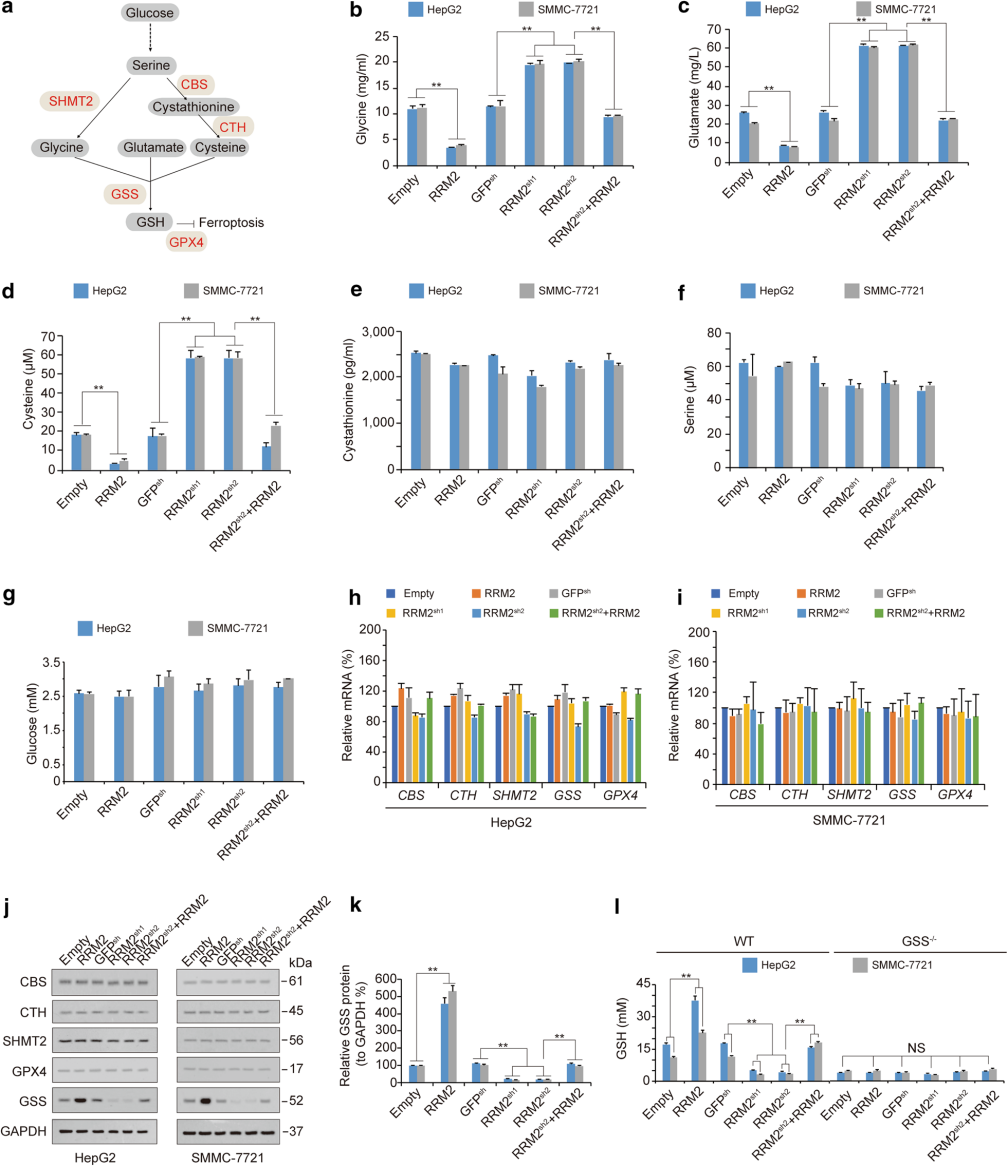
RRM2 upregulates GSH by sustaining GSS. **a** The ferroptosis-related metabolic axis from glucose to GSH. **b**–**g** The levels of glycine (**b**), glutamate (**c**), cysteine (**d**), cystathionine (**e**), serine (**f**) and glucose (**g**) were measured in HepG2 and SMMC-7721 cells with or without ectopically expression or knocked down of RRM2. **h**, **i** mRNA levels of *CBS*, *CTH*, *SHMT2*, *GSS* and *GPX4* were analyzed by qPCR in HepG2 (**h**) and SMMC-7721 cells (**i**) administered the indicated treatment. **j**, **k** Protein levels of CBS, CTH, SHMT2, GSS and GPX4 were analyzed by immunoblotting in HepG2 and SMMC-7721 cells (**j**). The level of RRM2 was normalized to that of GAPDH, and the normalized level of RRM2 in the untreated group was arbitrarily set to 100% (**k**). (**l**) GSH levels were measured in WT and GSS^−/−^ HepG2 and SMMC-7721 cells with or without RRM2 overexpression or knockdown, as indicated. The data are shown as the mean ± SD from three biological replicates. **P < 0.01 indicates statistical significance. Data from **b**−**i** and **k**−**l** were analyzed using one-way ANOVA

While GSH can be synthesized from glycine, cysteine and glutamate, glycine and cysteine can be produced by the metabolic axis from glucose to serine (Fig. 3a) [18, 49, 50]. To trace the target where RRM2 influences GSH activity, we examined glycine, glutamate, cysteine, cystathionine, serine and glucose levels before and after alteration of RRM2 expression in HepG2 and SMMC-7721 cells. The levels of glycine, glutamate and cysteine were reduced significantly by RRM2 overexpression (Fig. 3b–d), suggesting that RRM2 stimulates GSH synthesis by increasing the utilization of its raw materials. However, cystathionine, serine and glucose production were not influenced by RRM2 (Fig. 3e–g), further demonstrating that the target site of RRM2 is located downstream of glycine, glutamate and cysteine.

Next, we examined the enzymes that participate in GSH metabolism to investigate which is essential for RRM2 to regulate GSH. These enzymes include cystathionine beta-synthase (CBS), cystathionine gamma-lyase (CTH), serine hydroxymethyltransferase 2 (SHMT2), glutathione synthetase (GSS) and glutathione peroxidase 4 (GPX4) (Fig. 3a). Unfortunately, RRM2 influenced none of these enzymes at the mRNA level (Fig. 3h, i). However, only the protein level of GSS, which facilitates GSH synthesis from glycine, glutamate and cysteine, was positively modulated by RRM2 (Fig. 3j, k), suggesting that RRM2 regulates GSS at the protein level. Furthermore, the effects of RRM2 on elevating GSH were abolished when GSS was knocked out by CRISPR-Cas9 technology in both HepG2 and SMMC-7721 cells (Fig. 3l), demonstrating that GSS is a prerequisite for RRM2 to upregulate GSH in liver cancer cells.

### Degradation of GSS is facilitated following dephosphorylation of RRM2 under ferroptotic stress

**Fig. 4:**
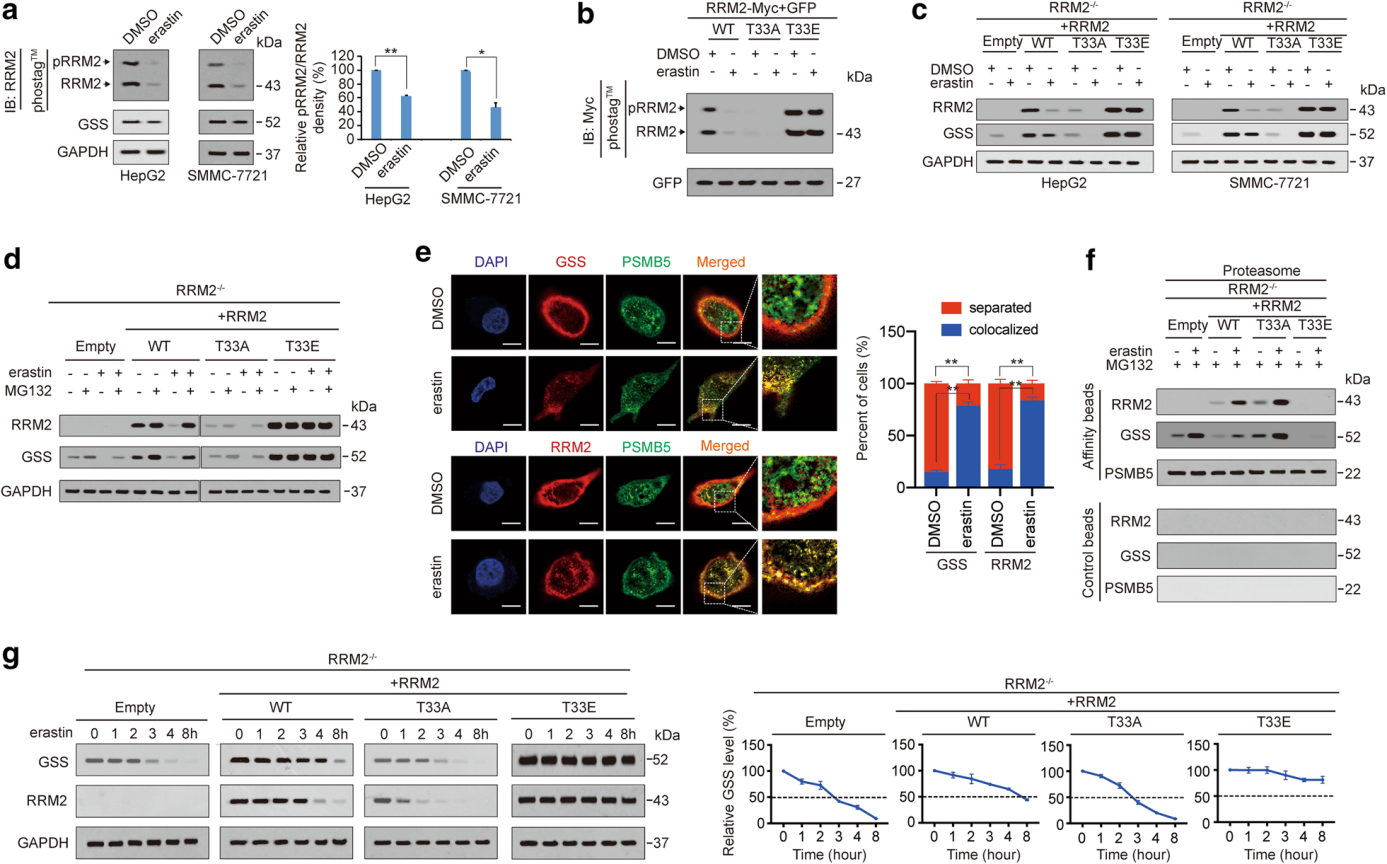
Phosphorylation of RRM2 at T33 protects GSS from proteasome degradation. **a** pRRM2 and RRM2 were measured by immunoblotting using anti-RRM2 antibodies following electrophoresis in gels containing Phos-tag™, while GSS was measured by immunoblotting using anti-GSS antibodies following electrophoresis in conventional gels. HepG2 and SMMC-7721 cells were cultured in the presence or absence of erastin (10 μM) for 24 h. Relative pRRM2/RRM2 ratios between groups were also calculated and graphed. **b** RRM2 was phosphorylated at T33. pRRM2 and RRM2 levels were measured by immunoblotting with anti-Myc antibodies following electrophoresis in Phos-tag™-containing gels of HepG2 cells ectopically expressing RRM2^WT^, RRM2^T33A^ or RRM2^T33E^ before and after treatment with erastin (10 μM) for 24 h. **c** RRM2 and GSS were measured by immunoblotting in RRM2^−/−^ HepG2 and SMMC-7721 cells ectopically expressing RRM2^WT^, RRM2^T33A^ or RRM2^T33E^ before and after treatment with erastin (10 μM) for 24 h. **d** RRM2 and GSS were degraded by proteasomes. RRM2 and GSS were measured by immunoblotting in HepG2 cells with the indicated treatments. Erastin and MG132 were treated at concentrations of 10 μM and 8 μM, respectively, for 24 h. **e** Colocalization of GSS (upper) or RRM2 (lower) with PSMB5 in HepG2 cells cultured in the presence or absence of erastin (10 μM, 24 h). Scale bar, 20 μm. **f** Association of RRM2, GSS and PSMB5 in proteasomes isolated from HepG2 cells cultured in the presence or absence of erastin (10 μM, 24 h) in the presence of MG132 (8 μM, 24 h). Samples from affinity or control beads were analyzed in parallel. **g** Erastin (10 μM, 24 h) chase of GSS and RRM2 in RRM2^−/−^ HepG2 and SMMC-7721 cells reconstituted with RRM2^WT^, RRM2^T33A^ or RRM2^T33E^. The levels of GSS were also normalized to those of GAPDH, and the normalized level of GSS in the 0 h group was arbitrarily set to 100%. The data are shown as the mean ± SD from three biological replicates (including IB). *P < 0.05, **P < 0.01 indicates statistical significance. Data from a were analyzed using a one-way ANOVA test. Data from e were analyzed using Student’s t test

Next, we investigated how RRM2 regulates GSS under ferroptotic stress. Phosphorylation of RRM2 at threonine 33 (pRRM2^T33^) influences its expression [51]. Hence, we first evaluated the phosphorylation status of RRM2 in HepG2 and SMMC-7721 cells using Phos-tag™ before and after treatment with erastin. Both the phosphorylated and total levels of RRM2 were reduced following erastin treatment, and the degree to which phosphorylation was reduced was more obvious than that of total RRM2 (Fig. 4a), suggesting that suppression of phosphorylation occurs prior to that of total RRM2. As expected, GSS was also reduced following erastin treatment (Fig. 4a), indicating that downregulation of GSS might be a result of dephosphorylation of RRM2.

Then, we replaced the T33 with alanine (A) and glutamic acid (E) to abolish and mimic phosphorylation of RRM2, respectively, and the T33A and T33E mutants of RRM2 were constructed. As expected, phosphorylation of RRM2 was lost and enhanced in T33A and T33E mutants, respectively, compared to that of the RRM2^Wild type (WT)^, suggesting that phosphorylation occurred at T33 (Fig. 4b). Reconstitution of RRM2^WT^ in RRM2^−/−^ HepG2 and SMMC-7721 cells resulted in an elevation of basal GSS protein expression. This reconstitution also reduced the level of GSS suppression following erastin treatment (Fig. 4c). However, the effects on GSS protein before and after reconstitution of RRM2^T33A^ were still comparable (Fig. 4c), suggesting that dephoshporylation impairs the ability of RRM2 to boost GSS expression. By contrast, RRM2^T33E^ not only elevated GSS protein at the basal level but also enabled GSS resistance to erastin (Fig. 4c), further supporting that phosphorylation of RRM2 at T33 is essential to sustain GSS protein expression. Similar to GSS, we found that phosphorylation at T33 is also critical to sustain the protein level of RRM2 itself (Fig. 4c). These results suggested that RRM2 and GSS proteins are regulated under similar mechanisms both at basal condition and under ferroptotic stress.

Because changes in phosphorylation are often followed by protein degradation in the proteasome [52, 53], we investigated whether RRM2 and GSS expressions are suppressed in a proteasome-dependent manner. Treatment with MG132, a proteasome inhibitor, slightly increased the basal levels of RRM2 and GSS proteins in RRM2^WT^-expressing HepG2 cells (Fig. 4d), suggesting that both proteins can be degraded by the proteasome. We also found that erastin-mediated suppression of RRM2 and GSS was completely reversed by MG132 in RRM2^WT^-expressing HepG2 cells (Fig. 4d), suggesting that proteasome degradation of RRM2 and GSS is enhanced under ferroptotic stress. Compared to RRM2^WT^, RRM2^T33A^ was unable to alleviate proteasome degradation of RRM2 and GSS. By contrast, RRM2^T33E^ almost completely prevented RRM2 and GSS from proteasome degradation both at basal levels and under ferroptotic stress (Fig. 4d), indicating that pRRM2^T33^ promotes RRM2 and GSS protein expression by inhibiting their proteasome degradation. By confocal experiments using PSMB5 (one activity site of the proteasome) as the marker to indicate the proteasome, RRM2 and GSS were found to be recruited to the proteasome following erastin treatment (Fig. 4e). In the isolated proteasome, we also found that erastin-stimulated RRM2 and GSS into the proteasome were negatively associated with the level of pRRM2^T33^ (Fig. 4f). The degradation rates of GSS and RRM2 were finally evaluated and we found that in addition to accelerating their own degradation, dephosphorylation of RRM2 at T33 also accelerated the degradation of GSS following erastin treatment (Fig. 4g). Overall, dephosphorylation of RRM2 at T33 is a prerequisite for proteasome degradation of RRM2 and GSS under ferroptotic stress.

### Dephosphorylation of RRM2 stimulates the RRM2–GSS interaction and their corecruitment to the proteasome

**Fig. 5:**
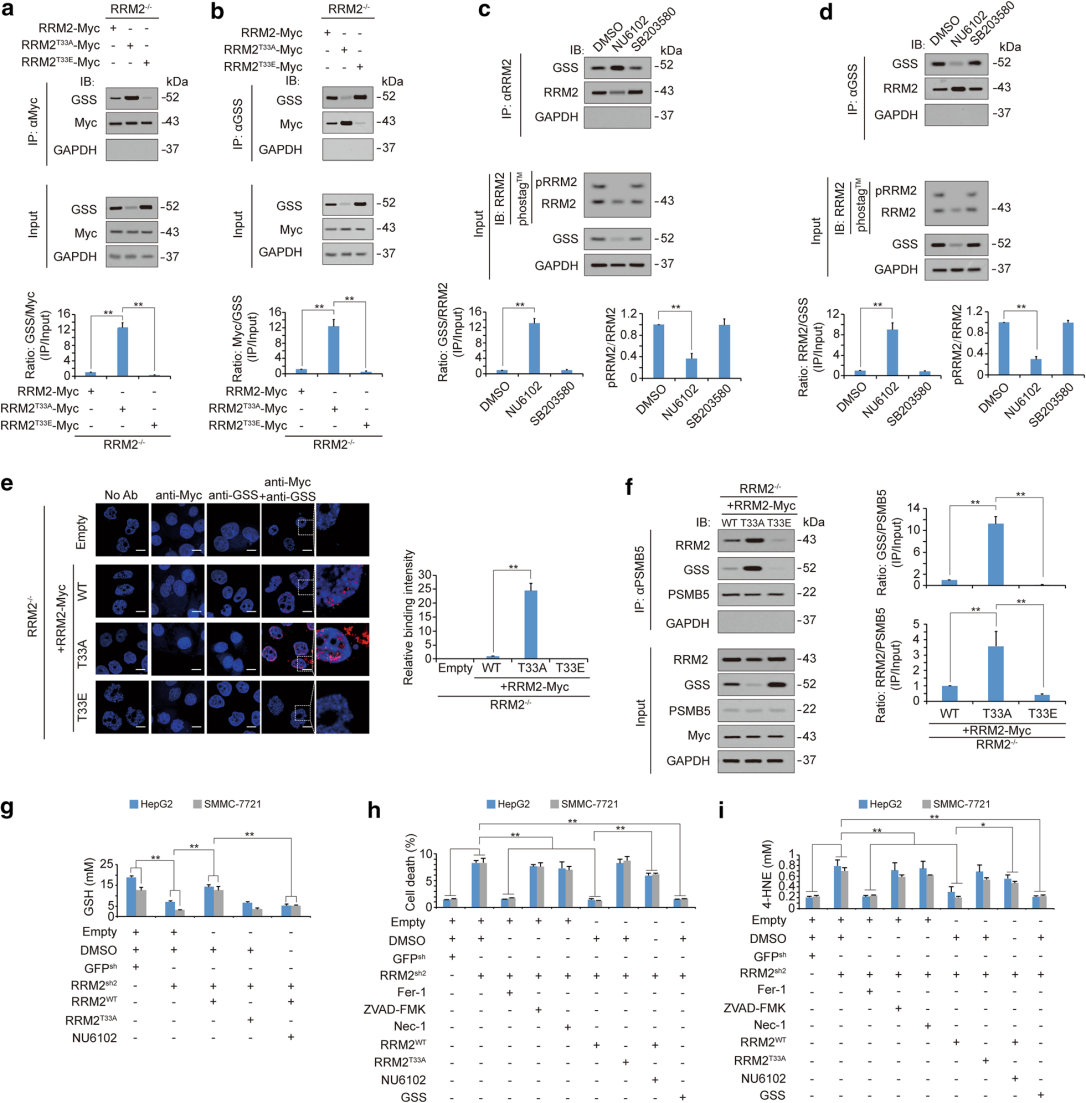
Dephosphorylation of RRM2 stimulates RRM2 binding with GSS to promote their corecruitment to the proteasome and subsequent activation of ferroptosis. **a**, **b** Reciprocal IP experiments of RRM2 and GSS in RRM2^−/−^ HepG2 cells reconstituted with RRM2^WT^, RRM2^T33A^ or RRM2^T33E^. The amount of proteins in the immunoprecipitates was normalized to that in whole-cell lysates (Input), and was graphed in the lower panel. **c**, **d** Reciprocal IP experiments for RRM2 and GSS in HepG2 cells with or without NU6102 (20 μM, 24 h) and SB203580 (10 μM, 24 h) treatment. The amount of protein in the immunoprecipitates was normalized to that in whole-cell lysates (Input), and was graphed in the lower panel. **e** Proximal protein ligation between endogenous GSS and the indicated exogenous RRM2-Myc, as measured by PLA in HepG2 cells expressing RRM2^WT^, RRM2^T33A^ or RRM2^T33E^. Scale bar, 20 μm. The PLA signals were also calculated and graphed, and the data from the “Empty” group were arbitrarily set to 1. **f** RRM2^WT^, RRM2^T33A^ or RRM2^T33E^ was expressed in reconstituted RRM2^−/−^ HepG2 cells. Immunoprecipitations were acquired with anti-PSMB5 antibodies and further analyzed by immunoblotting using anti-RRM2 and anti-GSS antibodies. GSS and RRM2 enrichment in the immunoprecipitates was calculated as the normalization to the levels in whole-cell lysates (input). **g**–**i** GSH (**g**), cell death (**h**) and 4-HNE (**i**) were measured in HepG2 and SMMC-7721 cells with or without RRM2 knockdown before they were further treated with Fer-1 (1 μM, 24 h), ZVAD-FMK (20 μM, 24 h), Nec-1 (20 μM, 24 h), or ectopically expressed GSS, RRM2^T33A^ or RRM2^WT^ in the presence or absence of NU6102 (20 μM, 24 h). The data are shown as the mean ± SD from three biological replicates (including IB). *P < 0.05, **P < 0.01 indicates statistical significance. The data from **a**–**d** and **f**–**i** were analyzed using one-way ANOVA

Because dephosphorylation of RRM2 triggers proteasome degradation of both RRM2 and GGS (Fig. 4), we wondered whether dephosphorylation of RRM2 facilitates the interaction between RRM2 and GSS. Reciprocal co-IP experiments demonstrated that among RRM2^WT^, RRM2^T33A^ and RRM2^T33E^, RRM2^T33A^ had the strongest interaction with GSS, while RRM2^T33E^ had the weakest interaction (Fig. 5a, b). At the pharmacological level, treatment of HepG2 cells with NU6102, a pRRM2^T33^ inhibitor [51] also resulted in increases in the RRM2–GSS interaction, while treating SB203580, a p38 inhibitor, exerted no such effects (Fig. 5c, d), suggesting that dephosphorylation of RRM2 enhances the RRM2–GSS interaction. The PLA confirmed that the RRM2–GSS interaction was enhanced following abolishment of RRM2 phosphorylation at T33 (Fig. 5e). As PSMB5 is a critical component of the proteasome [54, 55], we immuoprecipitated PSMB5 to test whether dephosphorylation of RRM2 influences corecruitment of GSS and RRM2 into the proteasome. Compared to RRM2^WT^ expression, RRM2^T33A^ expression reinforced not only the interaction between GSS and PSMB5, but also the interaction between RRM2 and PSMB5. By contrast, RRM2^T33E^ expression had the opposite effect (Fig. 5f). These results demonstrated that dephosphorylation of RRM2 enhances the RRM2–GSS interaction and their corecruitment into the proteasome.

### *Phosphorylation of RRM2 prevents ferroptosis *via* GSS to maintain GSH levels*

To further investigate whether phosphorylation of RRM2 prevents ferroptosis in liver cancer cells, GSH levels were first examined. We found that knocking down RRM2 reduced the GSH concentration, which could be reversed by ectopic expression of RRM2^WT^; however, this effect could not be reversed by RRM2^T33A^. The rescue effects of RRM2^WT^ were blocked when HepG2 and SMMC-7721 cells were treated with NU6102 (Fig. 5g), suggesting that phosphorylation of RRM2 is critical to maintain GSH. Ferroptosis was then tested, and we found that in addition to overexpressing GSS, knocking down RRM2 induced ferroptosis, and ferroptosis-associated 4-HNE elevation could only be reversed by ectopic expression of RRM2^WT^ (Fig. 5h, i), further demonstrating that phosphorylation of RRM2 at T33 prevents ferroptosis via GSS.

### Serum RRM2 is a novel biomarker of liver cancer

**Fig. 6:**
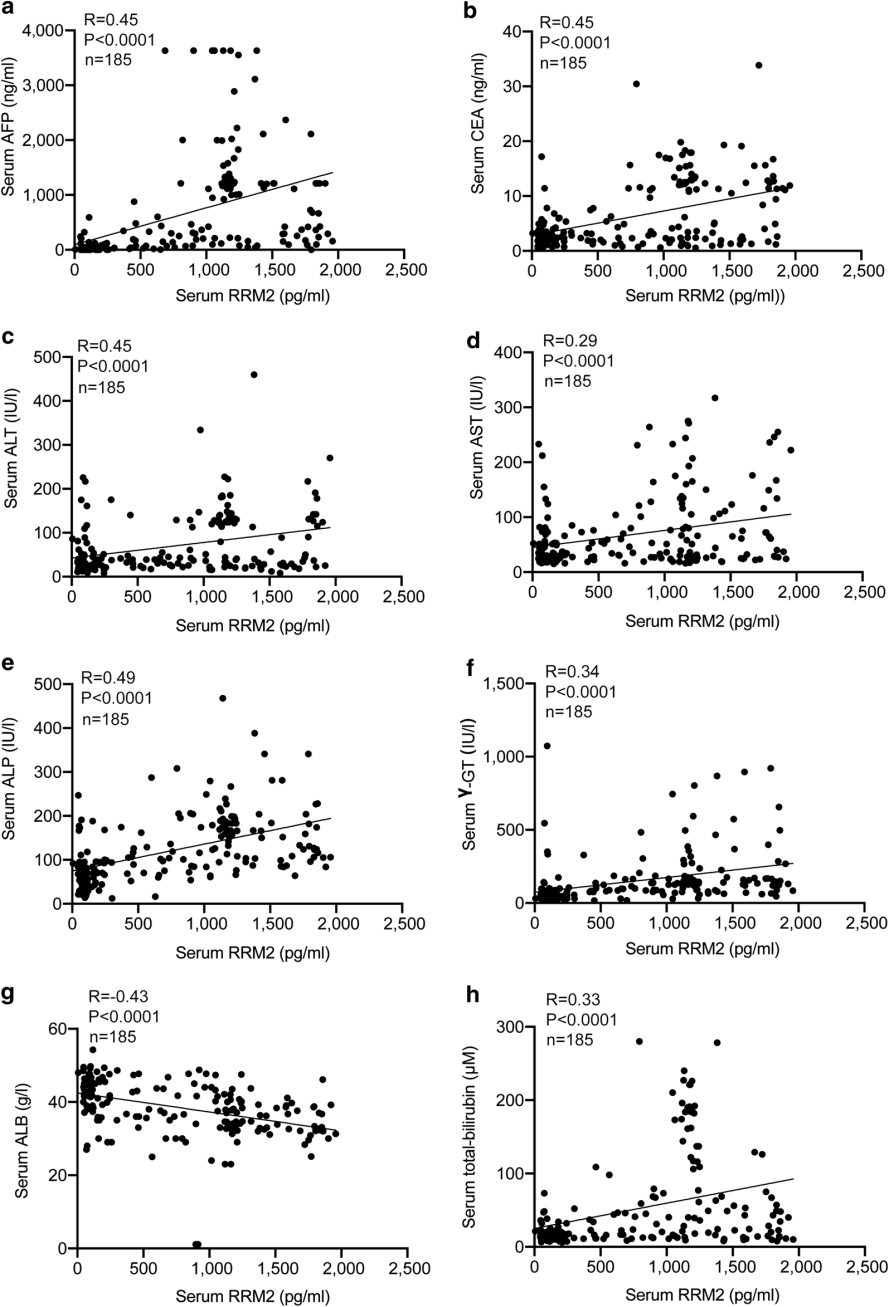
Relationship among serum RRM2, serum AFP and the parameters of liver function in liver cancer patients. The relationships between serum RRM2 levels and serum AFP (**a**), CEA (**b**), ALT(**c**), AST (**d**), ALP (**e**), γ-GT (**f**), ALB (**g**), and total bilirubin (**h**) in liver cancer patients are shown. Data from 185 liver cancer patients (from three biological replicates) were used to analyze the relationship using Spearman's rank correlation coefficient

**Fig. 7:**
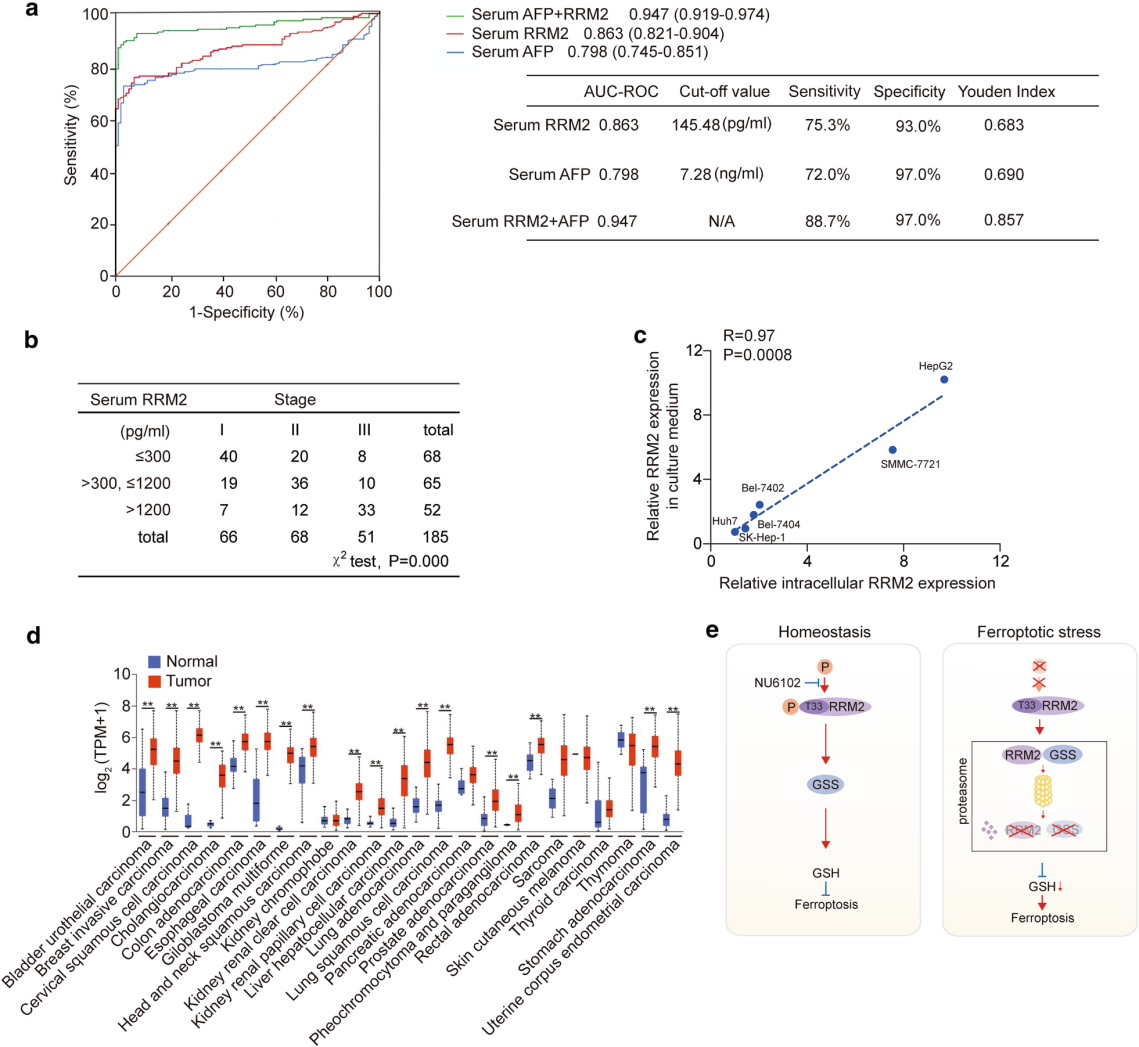
Diagnostic value of serum RRM2 in predicting liver cancer. **a** ROC curves for serum RRM2, AFP, and the combination of serum RRM2 and AFP for the discriminating patients with liver cancer from healthy individuals. The AUC value represents the combined effects of the sensitivity and specificity of single or combined biomarkers in the diagnosis of patients with liver cancer. All the data were obtained from three biological replicates. **b** Serum RRM2 is positively associated with tumor stage in liver cancer patients. The first quartile of serum RRM2 levels in liver cancer patients was no more than 300 pg/ml, the second quartile was from 300 to 1200 pg/ml, and the bottom quartile was more than 1200 pg/ml. Liver cancer patients were divided into three groups according to these quartiles. All the data were from three biological replicates. The associations between tumor stage and serum RRM2 in liver cancer patients were analyzed using the χ^2^ test. **c** Correlation between intracellular RRM2 and RRM2 in culture medium from liver cancer cell lines as analyzed by Spearman rank-correlation analysis. **d** RRM2 expression across multiple cancer types from the UALCAN database. TPM, transcriptions per million. **e** The model of the study. Under homeostasis, RRM2 is phosphorylated at the T33 site (can be dephosphorylated by NU6102) to sustain GSS and GSH levels and suppress potential ferroptosis. In the ferroptotic state, RRM2 is dephosphorylated and has increased affinity GSS, leading to the subsequent proteasomal degradation of both proteins. With such an effect, GSH eventually declines to facilitate ferroptosis. The data from a-c are shown from three biological replicates. Analysis of the receiver operator characteristics (ROC) and calculation of the area under the curve (AUC) was performed to find the diagnostic values of RRM2, AFP and the combination of RRM2 and AFP for the prediction of liver cancer. **P < 0.01 indicates statistical significance. Data from b were analyzed using the χ^2^ test. Data from c were analyzed by Spearman rank-correlation analysis. Data in d were analyzed using Student’s t test

Since RRM2 was specifically elevated in serum from liver cancer patients (Fig. 1c, d), we investigated whether serum RRM2 can be used as a diagnostic biomarker for liver cancer. Serum AFP is a classic tumor biomarker for liver cancer [56, 57]. Scatter distributions of serum RRM2 and serum AFP indicated a positive correlation between serum RRM2 and serum AFP (R = 0.45, P < 0.0001, Fig. 6a). Serum RRM2 was also positively correlated with serum carcinoembryonic antigen (CEA) (R = 0.45, P < 0.0001, Fig. 6b), a tumor biomarker for the digestive tract [56, 58]. Then, we investigated the relationship between serum RRM2 levels and other indicators related to liver function. The results showed that serum RRM2 levels were significantly correlated with alanine aminotransferase (ALT) (R = 0.45, P < 0.0001, Fig. 6c), aspartate aminotransferase (AST) (R = 0.29, P < 0.0001, Fig. 6d), alkaline phosphatase (ALP) (R = 0.49, P < 0.0001, Fig. 6e), gamma glutamyltranspeptidase (γ-GT) (R = 0.34, P < 0.0001, Fig. 6f), albumin (ALB) (R = -0.43, P < 0.0001, Fig. 6g), and total bilirubin (R = 0.33, P < 0.0001, Fig. 6h). The area under the receiver operating characteristic curve (AUC-ROC) analysis indicated that serum RRM2 (AUC: 0.863, 95% CI 0.821–0.904) was a better diagnostic marker of liver cancer than AFP (AUC: 0.798, 95% CI 0.745–0.851). The cutoff point that best predicted liver cancer was 145.48 pg/ml (Fig. 7a). AUC-ROC analysis also indicated that the combination of RRM2 with AFP to diagnose liver cancer (AUC: 0.947, 95% CI 0.919–0.974) was even better than either AFP or RRM2 alone, with a sensitivity of 88.7% and a specificity of 97.0% (Fig. 7a). In addition, higher serum RRM2 concentrations were significantly associated with higher tumor stage in liver cancer (Fig. 7b). These results indicate that RRM2 is a promising biomarker for liver cancer. Additionally, we found that the RRM2 concentration in the culture medium correlated well with the intracellular RRM2 levels in a series of established liver cancer cell lines (Fig. 7c), suggesting that the release of serum RRM2 might be positively correlated with its intracellular expression level in liver cancer tissues. Because RRM2 has roles in protecting liver cancer cells against ferroptosis, testing serum RRM2 might also be a promising method to predict ferroptosis resistance for ferroptosis-based treatments for liver cancer.

Finally, to further reveal the importance of RRM2 in other cancers, we evaluated the RRM2 expression pattern in a series of cancer types from the UALCAN database. RRM2 was also elevated in a series of tumor tissues compared to normal tissues from their corresponding organs (Fig. 7d), suggesting that similar to liver cancer, RRM2 exerts anti-ferroptotic activities in other cancers. However, serum RRM2 was elevated only in the patients with liver cancer among the cancer types we tested (Fig. 1c), which might be due to the mechanisms underlying how RRM2 release into the blood stream varies among different types of cancers. Such mechanisms will be investigated in our future study.

## Discussion

Ferroptosis is triggered following the accumulation of lipid peroxides [47]. However, GSH, which is involved in the antioxidant system, has the capacity to protect cancer cells against potential ferroptosis [18, 59, 60]. In our previous studies, we found that ferroptosis is more likely to be inhibited in liver cancer cells because the transcriptional signaling pathway controlled by HNF4A corresponds to a series of ferroptosis-resistant molecules [18]. In this study, we further clarified that high expression of RRM2 is another potential intracellular anti-ferroptotic event in liver cancer (Fig. 7e).

GSH is one of the most important antioxidants and protects cells against lipid peroxide damage [38, 61]. By utilizing GSH, ferroptosis is antagonized because lipid peroxides are reduced to their corresponding alcohols [9, 11]. It is also not difficult to understand that, exhausting GSH is a potential way to trigger ferroptosis [38, 62, 63]. GSH can be synthesized from three critical amino acids: cysteine, glutamate and glycine [64, 65]. Both glycine and cysteine can be produced by the metabolic axis from glucose to serine [49, 50]. Therefore, the metabolites and enzymes that convert serine to GSH are critical to sustain an anti-ferroptotic activity in cancer cells, especially those that are extremely metabolically active. For example, knockdown of CBS, the biosynthetic enzyme for cysteine in erastin-resistant cells causes ferroptotic cell death. In contrast, CBS overexpression confers ferroptosis resistance [66]. For GSS, one study reported that inhibiting GSS by solasonine, a compound isolated from *Solanum melongena*, elevates lipid ROS levels in HepG2 cells [67]. In the current study, we further uncovered that GSS is tightly regulated by RRM2, and dephosphorylation of RRM2 at T33 facilitates proteasome-mediated degradation of RRM2 and GSS by similar mechanisms (Fig. 7e). RRM2 is prone to be dephosphorylation under ferroptotic stress, thus also providing evidence to explain why the levels of RRM2 and GSH are simultaneously decline when ferroptosis is triggered. Interestingly, RRM2 has been reported to be sensitive to oxidative stress [68]. Under oxidizing conditions, RRM2 forms disulfide-bonded dimers that are more susceptible to proteolysis [68]. The process that induces ferroptosis is also a kind of oxidative stress; therefore, inhibiting proteolysis of RRM2 might be an effective way to prevent ferroptosis. In other words, cells can be sensitized to ferroptosis once RRM2 is degraded. Overall, RRM2 is a potential target for ferroptosis-based therapy to treat liver cancer.

The sensitivity of the classic liver cancer diagnosis marker AFP is less than 80% [69]. On some occasions, other diseases, such as hepatitis, cirrhosis, colorectal cancer and lung cancer, also present with elevated AFP [69]. New diagnostic biomarkers are needed to improve AFP diagnostic performance. In liver cancer, the GSH-related antioxidant system inhibits ferroptosis and provides energy for cell growth and metastasis [61, 70, 71]. Therefore, one could discover new diagnostic liver cancer biomarkers from the GSH synthesis pathway. Here, we found that RRM2 protects GSS from proteasome degradation, thus maintaining the GSH concentration to prevent damage from lipid peroxides. Although many tumor biomarkers linking apoptosis have been reported [72–74], one reflecting ferroptosis suppression was first reported by our lab in this study. Furthermore, we proved that the combining serum RRM2 and serum AFP resulted in a better diagnostic performance than using either RRM2 or AFP alone. Multibiomarker combined diagnosis for liver cancer is a future trend. For example, a microRNA panel developed by Zhou et al. provided a high diagnostic accuracy of liver cancer [75]. Moreover, six types of phospholipids also allowed for the confident determination of liver cancer [76]. Hence, we believe that compared to a single biomarker, diagnosis panels comprising multiple markers for liver cancer will significantly improve the sensitivity and specificity of liver cancer diagnoses in the future.

## Conclusion

In conclusion, we elucidated that RRM2 exerts anti-ferroptotic function in liver cancer cells by sustaining intracellular GSH by protecting GSS from degradation. We also preliminarily verified that serum RRM2 is a potential diagnostic biomarker for liver cancer. Together, proteins that protect against ferroptosis can be regarded as both targets and biomarkers for the diagnosis and treatment of liver cancer.

## Supplementary Information

**Additional file 1: Table S1.** Sequence of primers and sgRNA were listed.

## Data Availability

The data sets used and/or analyzed during the current study are available from the corresponding author on reasonable request.

## Abbreviations

GSH: Glutathione
RRM2: Ribonucleotide reductase regulatory subunit M2
ELISA: Enzyme linked immunosorbent assay
qPCR: Quantitative RT-PCR
IB: Immunoblotting
IHC: Immunochemistry
GSS: Glutathione synthetase
IF: Immunofluorescence
co-IP: Co-immunoprecipitation
PLA: Proximal ligation analysis
AUC-ROC: Area under the receiver operating characteristic curve
AFP: Alpha-fetoprotein
CEA: Carcinoembryonic antigen
ALT: Alanine aminotransferase
AST: Aspartate aminotransferase
ALP: Alkaline phosphatase
γ-GT: Gamma glutamyltranspeptidase
ALB: Albumin
ROS: Reactive oxygen species
TF: Transcription factor
HIC1: Hypermethylated in cancer 1
HNF4A: Hepatocyte nuclear factor 4 alpha
MT-1G: Metallothionein-1G
RR: Ribonucleotide reductase
CT: Computed tomography
MRI: Magnetic resonance imaging
TMT: Tandem mass tags
TCGA: The Cancer Genome Atlas
CBS: Cystathionine beta-synthase
CTH: Cystathionine gamma-lyase
SHMT2: Serine hydroxymethyltransferase 2
GPX4: Glutathione peroxidase 4
