# Supplementary material for: RRM2 protects against ferroptosis and is a tumor biomarker for liver cancer
Source: Cancer Cell Int. 2020 Dec 7;20:587. doi: 10.1186/s12935-020-01689-8 (PMC7720568; doi:10.1186/s12935-020-01689-8)
Supplement: Supplementary file 1 — Additional file 1: Table S1. Sequence of primers and sgRNA were listed. [file 12935_2020_1689_MOESM1_ESM.docx]

**Table S1. Sequence of primers and sgRNA were listed.**

| Primers used for qPCR |  | |  | | |  | | |  |  |
| --- | --- | --- | --- | --- | --- | --- | --- | --- | --- | --- |
| Name | | 5'-3' | |  | | |  | | |  |
| CBS-F | | tcctagaccagtaccgcaacgcca | | | | | | | | |
| CBS-R | | caatgatcctgcatccaggacact | | | | | | | | |
| CTH-F | | attgacattgaaggctgtgcacat | | | | | | | | |
| CTH-R | | agacaccaggcccattacaacatc | | | | | | | | |
| SHMT2-F | | agttgctgcagagggagaaggaca | | | | | | | | |
| SHMT2-R | | gcagctcaatttcatccaccacct | | | | | | | | |
| GSS-F | | cttcaacctgctagtggatgctgt | | | | | |  | | |
| GSS-R | | tggaacatgtagtctgagcgattc | | | | | |  | | |
| GPX4-F | | agtggatgaagatccaacccaagg | | | | | |  | | |
| GPX4-R | | gggccacacacttgtggagctaga | | | | | | | | |
| GAPDH-F | | ATCATCCCTGCCTCTACTGG | | | | | |  | | |
| GAPDH-R | | GTCAGGTCCACCACTGACAC | | |  | | |  | | |
|  |  | |  | | |  | | |  |  |
| sgRNA sequence |  | |  | | |  | | |  |  |
| Name | | 5'-3' | | |  | | |  | | |
| RRM2-KO-F | | CACCGGATCCTCCTCGCGGTCTTGC | | | | | | | | |
| RRM2-KO-R  GSS-KO-F  GSS-KO-R | | AAACGCAAGACCGCGAGGAGGATCC  CACCGGATGGACTTCAACCTGCTAG  AAACCTAGCAGGTTGAAGTCCATCC | | | | | | | | |
|  | |  | | | | | | | | |
| Primers used for plasmid construction | |  | | | | | | | | |
| Name | | 5'-3' | | | | | | | | |
| WT-RRM2-Myc-F | | atgcGGTACCatgctctccctccgtgtcccgc | | |  |  |  |  |  |  |
| WT-RRM2-Myc-R | | atgcggatccttacagatcttcttcagaaataagtttttgttcgaagtcagcatccaaggtaa | | |  |  |  |  |  |  |
| T33A-RRM2-F | | ccgccggccctgagcgggacccgcg | | |  |  |  |  |  |  |
| T33A-RRM2-R | | gctcagggccggcggcgcgttctccttgtcgaccaagctg | | |  |  |  |  |  |  |
| T33E-RRM2-F | | ccgccggccctgagcgggacccgcg | | | | | | | | |
| T33E-RRM2-R | | gctcagggccggcggctcgttctccttgtcgaccaagctg | | | | | | | | |
|  |  | | | | | | | |  |  |
|  |  | | | | | | | |  |  |
|  |  | | | | | | | |  |  |
|  |  | |  | | |  | | |  |  |
|  |  | |  | | |  | | |  |  |
